# The Role of BAG3 Protein Interactions in Cardiomyopathies

**DOI:** 10.3390/ijms252011308

**Published:** 2024-10-21

**Authors:** Hui-Qi Qu, Ju-Fang Wang, Alexandre Rosa-Campos, Hakon Hakonarson, Arthur M. Feldman

**Affiliations:** 1 The Center for Applied Genomics, Children’s Hospital of Philadelphia, Philadelphia, PA 19104, USA; quh@chop.edu; 2 Department of Medicine, Division of Cardiology, The Lewis Katz School of Medicine at Temple University, Philadelphia, PA 19140, USA; wangj25@chop.edu (J.-F.W.); arthur.feldman@tuhs.temple.edu (A.M.F.); 3 Proteomics Facility, Sanford-Burnham-Presby Medical Discovery Institute, La Jolla, CA 92037, USA; arosacampos@sbpdiscovery.org; 4 Division of Human Genetics, Division of Pulmonary Medicine, Children’s Hospital of Philadelphia, Philadelphia, PA 19104, USA; 5 Department of Pediatrics, The Perelman School of Medicine, University of Pennsylvania, Philadelphia, PA 19104, USA; 6 Faculty of Medicine, University of Iceland, 102 Reykjavík, Iceland

**Keywords:** apoptosis, BAG3, bioID, cardiomyopathy, in vitro

## Abstract

Bcl-2-associated athanogene 3 (BAG3) plays an important function in cellular protein quality control (PQC) maintaining proteome stability. Mutations in the *BAG3* gene result in cardiomyopathies. Due to its roles in cardiomyopathies and the complexity of BAG3–protein interactions, it is important to understand these protein interactions given the importance of the multifunctional cochaperone BAG3 in cardiomyocytes, using an in vitro cardiomyocyte model. The experimental assay was conducted using high pressure liquid chromatography coupled to tandem mass spectrometry (LC-MS/MS) in the human AC16 cardiomyocyte cell line with BioID technology. Proteins with BAG3-interaction were identified in all the 28 hallmark gene sets enriched in idiopathic cardiomyopathies and/or ischemic disease. Among the 24 hallmark gene sets enriched in both idiopathic cardiomyopathies and ischemic disease, 15 gene sets had at least 3 proteins with BAG3-interaction. This study highlights BAG3 protein interactions, unveiling the key gene sets affected in cardiomyopathies, which help to explain the molecular mechanisms of the cardioprotective effects of BAG3. In addition, this study also highlighted the complexity of proteins with BAG3 interactions, implying unwanted effects of BAG3.

## 1. Introduction

BAG cochaperone 3 (BAG3), also known as Bcl-2-associated athanogene 3, is a multi-functional protein, which is expressed ubiquitously in animals, and homologs have been reported in plants [1]. BAG3 was first recognized for its ability to bind to Bcl2 with subsequent inhibition of apoptosis while other studies have found that it supports a diverse array of cellular functions including autophagy [2], excitation–contraction coupling, mitochondrial function [3], and the integrity of the sarcomere [4]. BAG3 has been recognized as playing a critical role in cellular protein quality control (PQC) to maintain the health of the proteome [5]. Mutations in the *BAG3* gene result in both dilated cardiomyopathy (DCM) [6] and peripartum cardiomyopathy (PPCM) [7] in a dominant inheritance model. A critical role of BAG3 in cardiomyocytes involves the maintenance of mitochondrial homeostasis that is mediated by both heat shock protein 70 (Hsp70) and the small heat shock proteins HspB6 and HspB8 [8]. BAG3 also has anti-apoptotic activity by binding Bcl-2 [9]. BAG3 has four protein binding domains, including (1) one WW (Trp–Trp) domain, binding with proteins in signal transduction processes [10], e.g., the PDZ domain containing guanine nucleotide exchange factor 2 (PDZGEF2) to promote cell adhesion [11]; (2) two IPV (Ile–Pro–Val) motifs, binding with small heat shock proteins (sHsps) HspB6/HspB8 [12]; (3) one proline-rich repeat (PXXP) region, binding with SH3 (Src homology 3) motifs, e.g., in phospholipase C-γ (PLC-γ) that also serves as an attachment site for the dynein motor transport of misfolded proteins to the peri-nuclear aggresomes [13,14]; and (4) one BAG domain, binding with Hsp70 and Bcl-2 [12,13,14].

While it is true that BAG3 is recognized for its multifunctionality and its role has been extensively studied, there is still much to uncover about its complex interactions and functions, especially in the context of different diseases. For instance, while the association of BAG3 with both cardiomyopathy and Parkinson’s disease (PD) has been reported, the genetic associations are in the opposite directions, i.e., the risk allele in DCM is protective against PD. The DCM-associated SNP rs2234962 [15,16] is in tight linkage disequilibrium with the PD-associated SNP rs72840788 (r^2^ = 1 in European populations) [17,18]. We advocate for a greater focus on the intricate BAG3 interaction network, as a reductionist approach may fail to capture the subtleties and multifaceted nature of BAG3’s role. Due to the complexity of BAG3–protein interactions, it is useful to gain a better understanding of the specific proteins with which BAG3 participates in its activity as a multifunctional cochaperone by using an in vitro cell model to better understand BAG3 binding with other cellular proteins. For this purpose, we performed a proteomics study using the BioID proximity-dependent biotinylation method to identify proteins that interact with BAG3, particularly those from the gene sets with expression levels correlated with cardiomyopathies. BioID is a unique technology to screen for protein interactions in living cells [19]. In addition to direct protein interactions, BioID is able to identify weak or transient interactions, as well as proteins in close proximity. This discovery experiment was carried out to identify potentially novel proteins that bind to or interact with BAG3.

## 2. Results

### 2.1. Gene Expression in Idiopathic and Ischemic Cardiomyopathies

**Figure 1:**
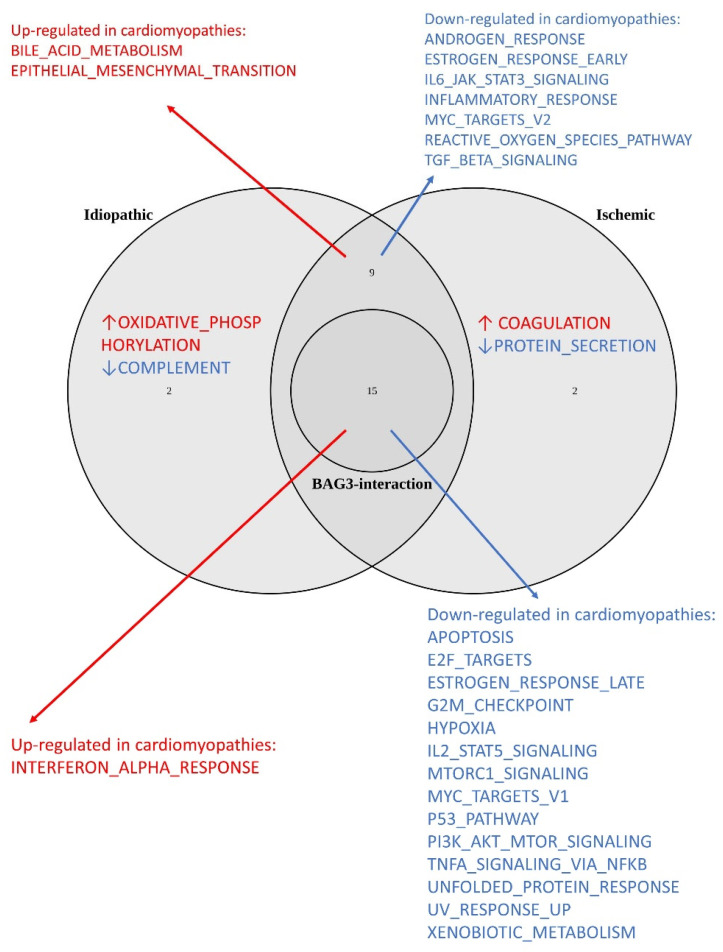
Venn plot of hallmark gene sets enriched in cardiomyopathies and those with BAG3 interactions. The central circle represents 15 cardiomyopathy-related gene sets containing at least three BAG3-interacting proteins. Red arrows indicate upregulated gene sets, while blue arrows indicate downregulated gene sets.

Among the 50 hallmark gene sets, 26 showed significance in idiopathic CM, and 26 showed significances in ischemic CM (Table 1, Figure 1). From these gene sets, 24 gene sets were significant in both idiopathic and ischemic CMs, while the other 4 gene sets had significances in only idiopathic or ischemic CMs. For the latter four gene sets, the same direction trend in enrichment was observed in the other type of CM. GO, KEGG, and Reactome gene set analyses are presented in Tables S1–S3. In particular, *BAG3* levels were decreased in both idiopathic (fold change = 0.646, adjusted *p*-value = 9.60 × 10^−6^) and ischemic (fold change = 0.633, adjusted *p*-value = 9.77 × 10^−6^) cardiomyopathies. WGCNA reveals that gene modules containing *BAG3* are downregulated in both idiopathic and ischemic CM (Figures S1 and S2).

### 2.2. BAG3–Protein Interactions

In total, 387 proteins were identified with significant adjusted *p*-values and positive BAG3–protein interactions (Table S4). Among these proteins, the *BRD4*, *CAST*, and *KLF6* genes show co-expression with *BAG3* in idiopathic CM based on WGCNA; the genes *ARFGAP3*, *RBM34*, *BAG3*, *BCCIP*, *BID*, *DNTTIP2*, *EBNA1BP2*, *EIF4A3*, *EIF5*, *ENAH*, *FOSL2*, *HSPA6*, *PRPF4*, *PUS7*, *RND3*, *SFPQ*, *SGK1*, *U2AF2*, and *ZMYND8* show co-expression with *BAG3* in ischemic CM based on WGCNA. BAG3-interaction were identified in each of the 28 hallmark gene sets enriched in the idiopathic and/or ischemic cardiomyopathies. Among the 24 hallmark gene sets enriched in both idiopathic and ischemic cardiomyopathies, 15 gene sets had at least 3 proteins with BAG3 interaction (Table 2, Figure 1).

## 3. Discussion

This study identified 387 proteins with direct or indirect BAG3 interactions. The mRNA levels in cardiomyocytes altered in DCM, as shown in the single-cell RNA-seq study by Koenig et al. [39], are annotated in Table S4. Among these proteins, heat shock 70 kDa protein 6 (HSPA6) [40] and Hsp70-binding protein 1 (HSPBP1) [41] encode a Hsp70 protein and regulate Hsp70 function, respectively. Bcl-2-associated transcription factor 1 (BCLAF1) activates the p53 pathway and induces apoptosis [42] and may contribute to myocardial reperfusion injury [43]. BCL2/adenovirus E1B 19 kDa protein-interacting protein 3-like (BNIP3L) activates the ER and mitochondrial cell death pathways and induces cardiomyopathy [44]. In addition, we observed 15 gene sets with at least 3 BAG3-interacting proteins further supporting the important role of BAG3 in the biology of the heart (Table 2).

**Figure 2:**
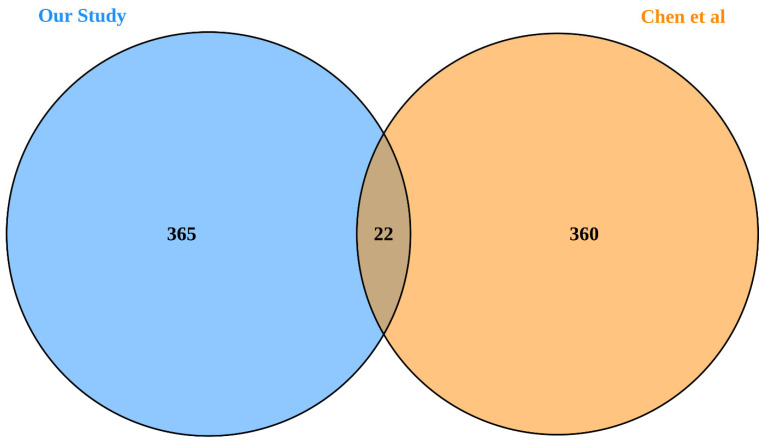
Venn plot of proteins identified in our study compared to those reported by Chen et al. [45].

In a previous study, Chen et al. identified 382 BAG3-interacting proteins in cancer cell lines using stable isotope labeling with amino acids in cell culture (SILAC) combined with mass spectrometry (MS) [45]. Of the 387 proteins identified in our study, 22 overlapped with those reported by Chen et al. (Figure 2). Given the approximately 20,000 protein-coding genes in the human genome, this overlap is highly statistically significant and unlikely to occur by chance alone (*p* = 3.69 × 10^−7^), providing mutual validation between these two studies. Among the 360 proteins identified by Chen et al. but not replicated in our study, 250 genes were detected in heart tissue by Hannenhalli et al. [46]. In contrast, 365 of the proteins identified in our study have not been reported by Chen et al. Of these, 295 genes were detected in heart tissue by Hannenhalli et al. [46], suggesting that our cell model is more informative for understanding heart-specific gene expression.

### 3.1. Proteins with Roles in Cell Cycle

Cardiomyocytes are taken as terminally differentiated and do not proliferate after birth [47]. In a recent study, a proliferative burst in preadolescent mice was observed [48]. However, further experimental validation is needed, as this finding has not yet been replicated by other researchers. If the same phenomenon is true in humans, the highlighted cell cycle proteins in this study may be implied for early intervention for cardiomyopathies, particularly for a BAG3-based therapy. Adult cardiomyocytes also exhibit a dynamic range of cell cycle activity under various physiological and pathological conditions, e.g., in pathologic myocardial hypertrophy [49].

BAG3 interaction was identified in 31 gene proteins from four important gene sets in the cell cycle, including MYC_TARGETS_V1, E2F_TARGETS, G2M_CHECKPOINT, and P53_PATHWAY. These four gene sets were downregulated in both idiopathic and ischemic cardiomyopathies. MYC, E2F, and p53 are important regulators of cell-cycle progression [50]. We observed BAG3 interaction of 13 proteins involving MYC signaling, including target variant 1 proteins, 11 proteins in E2F signaling molecules, 9 proteins in cell cycle G2/M checkpoint, and 3 proteins in the p53 pathway. The MYC_TARGETS_V1 genes are regulated by MYC [51] and are involved in cell-cycle progression and cell proliferation [52]. c-Myc plays an essential role in signaling DNA damage-induced apoptosis through the control of the p53 tumor suppressor protein [20]. Activation of the p53 pathway promotes cell cycle arrest to allow DNA repair or apoptosis for cells with serious DNA damage [23]. The G2/M checkpoints prevent DNA damaged cells from entering mitosis [22]. Acting through the E2F targets, E2F integrates cell cycle progression with DNA repair, replication, and G2/M checkpoints [21].

The proteins with BAG3-interaction in these gene sets also harbor opportunities for small molecular therapies. Deficiency of the MYC target gene, phosphoglycerate kinase 1 (*PGK1*), from genetic mutations, may cause myopathy [53]. The protein encoded by *PGK1* catalyzes the production of adenosine 5′-triphosphate (ATP) and its activity is regulated by ATP [54]. Biallelic deficiency of the MYC target gene—complement component 1 Q subcomponent-binding protein, mitochondrial (*C1QBP*)—causes mitochondrial respiratory-chain deficiencies and severe cardiomyopathy [55]. Copper supplementation has been shown to up-regulate the function of the *C1QBP* protein and may improve cardiac function [56].

### 3.2. Interactions with Proteins Involved in Apoptosis and Cell Damage Responses

In addition to the cell cycle proteins, BAG3 interacts with proteins in a number of gene sets downregulated in cardiomyopathies, including HALLMARK_APOPTOSIS, HALLMARK_HYPOXIA, HALLMARK_UV_RESPONSE_UP, HALLMARK_XENOBIOTIC_METABOLISM, HALLMARK_UNFOLDED_PROTEIN_RESPONSE, HALLMARK_PROTEIN_SECRETION, HALLMARK_COMPLEMENT, HALLMARK_TNFA_SIGNALING_VIA_NFKB, HALLMARK_MTORC1_SIGNALING, HALLMARK_ESTROGEN_RESPONSE_LATE, HALLMARK_PI3K_AKT_MTOR_SIGNALING, and HALLMARK_IL2_STAT5_SIGNALING. BAG3 interactions with apoptosis processes, responses to DNA damage and oxidative stress, and cell repairs were highlighted.

It has been suggested that cardiomyocyte apoptosis may contribute to myocardial reperfusion injury [57,58] and cardiomyopathies [59,60]. Currently, there is controversial evidence regarding the involvement of apoptosis in cardiomyopathies. Narula et al. [24] described an interruption in the apoptosis cascade, including nuclear fragmentation and condensation in cardiomyocytes, terming it ‘apoptosis-interruptus’. Conversely, our studies and others suggest that apoptosis plays a critical role in heart failure [61].

The gene expression data of cardiomyopathies show that the apoptosis pathway is downregulated in both idiopathic and ischemic cardiomyopathies. Cardiomyocytes with interrupted apoptosis may undergo necrosis. BAG3 binds to the Bcl-2 Homology 4 (BH4) domain of Bcl-2 to inhibit mitochondrial-dependent (intrinsic) apoptosis and, thus, protecting from cell death. Nine proteins in the APOPTOSIS gene set were identified for BAG3 interaction in this study, including three caspases CASP1, CASP3, and CASP4.

Hypoxia significantly alters myocardial gene expression, mediated by hypoxia inducible factor 1 subunit alpha (HIF1A) [62]. Hypoxia has been shown to induce dilated cardiomyopathy in chick embryos [63]. Overexpression of BAG3 has been shown to attenuate hypoxia-induced cardiomyocyte apoptosis [25]. DNA damage induces apoptosis and cardiomyopathy [27], whereas HALLMARK_UV_RESPONSE_UP genes (i.e., up-regulated genes in response to UV), play major roles in DNA damage repair [64]. Moreover, Xenobiotic toxicity causes cardiomyopathy through oxidative stress [26] resulting in dysregulated gene expression.

The unfolded protein response (UPR) decreases global protein synthesis, increases endoplasmic reticulum (ER)-associated degradation of misfolded proteins, and activates protein-folding in ER [28]. BAG3 serves as a cochaperone with Hsp70 and is involved in a wide range of protein folding processes, including the refolding of misfolded proteins [65]. The protein secretion pathway acts on protein folding, post-translational modifications (PTMs), and protein trafficking [66], and it is important in cardiac repair [67].

TNF-α [68] and complement activation [69] have been suggested as contributing factors to myocardial reperfusion injury and cardiomyopathy. However, complement activation contributes to tissue repair [70]. The genes encoding components of the complement system are downregulated in idiopathic and ischemic cardiomyopathies, which is in consistent with the recognition that long-term effects of complement inhibitors may be detrimental [71]. While NF-κB signaling may be cardioprotective in hypoxic or ischemic myocardial injury [29], it has also been shown to mediate chronic inflammation [72]. The mTORC1 signaling regulates cell growth and metabolism [30] and mediates adaptive cardiac hypertrophy [31]. Its inhibition increases overall protein degradation by the ubiquitin proteasome system [73] and may attenuate cardiac remodeling and heart failure [31]. The late estrogen response pathway may prevent apoptosis and necrosis of cardiac and endothelial cells [32]. Genes upregulated by activation of the PI3K/AKT/mTOR pathway include a major intracellular network that leads to cell proliferation [33] and inhibits cardiomyocyte apoptosis [34]. IL2_JAK_STAT5_SIGNALING genes are up-regulated by STAT5 in response to IL2 stimulation and modulate CD4+ Th cell differentiation [35], and upregulate the expression of c-Myc, BCL-2, and BCL-x further exacerbating the inflammatory response [36].

### 3.3. Interactions with Proteins Upregulated in Cardiomyopathies

In contrast to the above gene sets, the HALLMARK_INTERFERON_ALPHA_RESPONSE and HALLMARK_OXIDATIVE_PHOSPHORYLATION gene sets are both up regulated in cardiomyopathies. Interferon inhibits cardiac cell function in vitro [37] and interferon treatment has been shown to produce cardiotoxicity [38]. The heart is in high demand of energy by oxidative phosphorylation, where defective oxidative phosphorylation may cause cardiomyopathy [74]. Oxidative phosphorylation genes are upregulated in cardiomyopathies, which may cause overproduction of reactive oxygen species (ROS) [75]. BAG3 interactions with these upregulated genes in cardiomyopathies are interesting as this could ameliorate the cardioprotective effects of BAG3. Adjunct therapy targeting these proteins may, thus, improve the therapeutic effects of BAG3. The NADH dehydrogenases, i.e., NDUFB3 (NADH dehydrogenase 1 beta subcomplex subunit 3), NDUFB6 (NADH dehydrogenase 1 beta subcomplex subunit 6), and NDUFV1 (NADH dehydrogenase flavoprotein 1, mitochondrial), can be inhibited by metformin. The potential heart protective effects by metformin are gaining more research attention [76].

## 4. Research Design and Methods

Cell experimental assay: The cell model used in this study was the human AC16 cardiomyocyte cell line derived from adult human ventricular heart tissues (SCC109, Sigma-Aldrich, St. Louis, MO, USA). The cells were treated and analyzed based on five different conditions: (1) BAG3_Biotin: Cells transfected by plasmid expressing BioID-BAG3 for 48 h, then receive Biotin treatment for 16 h; (2) BioID: Cells transfected by plasmid expressing BioID vector only for 48 h, then receive Biotin treatment for 16 h; (3) BAG3 (to correct protein over-expression by BAG3): Cells transfected by plasmid expressing BioID-BAG3, without followed Biotin treatment; (4) AC16_Biotin (to correct protein-Biotin interaction): Cells receive Biotin treatment for 16 h; (5) AC16: Cells only. Three replicates were performed simultaneously for each treatment condition. Details of *BAG3* construct delivered using adeno-associated virus (AAV) vector has been described in our previous study [77]. The BioID experimental assay was conducted using high pressure liquid chromatography coupled to tandem mass spectrometry (LC-MS/MS) previously described through a collaboration with the Proteomics Facility, Sanford-Burnham-Presby Medical Discovery Institute, La Jolla, CA, USA [49].

Gene expression data: Gene expression data in tissue samples from left ventricular myocardium in two disease conditions, idiopathic and ischemic cardiomyopathies, were made available by Hannenhalli et al. [46]. The study included 16 controls, 86 idiopathic, and 108 ischemic cardiomyopathies. The heart tissue was snap-frozen at time of cardiac transplantation. The gene expression assay was based on data generated using the Affymetrix Human Genome U133A Array. The data analysis was performed by the GEO2R (https://www.ncbi.nlm.nih.gov/geo/info/geo2r.html, accessed on 9 October 2024). *p* values were adjusted by the Benjamini and Hochberg false discovery rate method. The data are publicly available at the NCBI Gene Expression Omnibus (GEO) database (https://www.ncbi.nlm.nih.gov/geo/query/acc.cgi?acc=GSE5406, accessed on 9 October 2024).

Gene Set Enrichment Analysis (GSEA) was performed by the GSEA v4.3.2 software (Broad Institute of MIT and Harvard, MA, USA) based on the Molecular Signatures Database (MSigDB) [78] hallmark [51], Gene Ontology (GO) [79], Kyoto Encyclopedia of Genes and Genomes (KEGG) [80], and Reactome [81] gene set collections. FDR corrected *p*-values < 0.05 were considered statistically significant. The Weighted Gene Co-expression Network Analysis (WGCNA) analysis was performed using the WGCNA R package [82,83].

Data analysis of BAG3–Protein interactions: The BioID data were analyzed using MSstats package v4.12.1 from R Bioconductor [84]. By comparing the groups of BAG3_Biotin vs. BioID, all proteins with adjusted *p*-value < 0.05 by the Benjamini and Hochberg false discovery rate method were identified. The effect sizes of BAG3–protein interactions were corrected by [log2FC(BAG3_Biotin vs. BioID)]-[log2FC(AC16_Biotin vs. AC16)]-[log2FC(BAG3 vs. BioID)], i.e., BAG3–protein interactions being corrected for biotin–protein interactions and BAG3-increased protein levels.

## 5. Conclusions

This study utilized the relatively new technique of BioID to identify interactions between BAG3 and various gene sets. The goal was to uncover unique proteins and protein pathways that might contribute to disease when BAG3 levels are under-expressed. Such under-expression can occur due to loss-of-function mutations caused by truncations, deletions, or unique mutations secondary to single nucleotide variants. These variants may change an amino acid, insert an amino acid, or alter the reading frame, resulting in truncation. Not unexpectedly, we identified a plethora of associated proteins. In view of the small size of BAG3, the limited number of binding sites, and the somewhat focused activities of the protein, the number of intersecting proteins identified by this new technique represents an overabundance of detected proteins. However, the fact that many of the observed proteins have been associated with BAG3 in various animal studies raises the larger question of how a single protein might interact with a large number of target proteins. The fact that virtually every known protein interaction with BAG3 has been detected by the BioID assay suggests that enhancing the assay’s sensitivity might allow for a more nuanced approach to this question. Additionally, new means of discriminating interactome data, which remain proprietary, could be useful.

In conclusion, this study highlights the observed interactions of BAG3 with key gene sets that are affected in cardiomyopathies, thereby unveiling some of the molecular mechanisms involved with the cardioprotective effects of BAG3. In addition, this study also highlights the complexity of proteins with BAG3 interactions, implying unwanted effects of BAG3. Adjunct therapy to address unwanted effects of BAG3 may be indicated, such as the use of metformin to inhibit the consequences of NADH dehydrogenase activation. A limitation of this study is the AC16 cell model, which is an immortalized cell line with passages over numerous generations [85], which may not represent the actual cardiomyocytes in human heart. Although AC16 cells are derived from human ventricles, they exhibit several key limitations: (1) they do not exhibit contractile activity, (2) they are highly proliferative, and (3) the sarcomere is not organized in AC16 cells. While AC16 cells differ from primary cardiomyocytes in these aspects, they remain a widely used in vitro model for studying human cardiomyocyte-related processes due to their human origin and ability to express cardiac-specific markers. The primary objective of our study was to investigate the complex BAG3 interaction network and identify its interacting proteins, so the aforementioned limitations do not significantly impact our findings. Further study using different cell models, in particular specialized cardiomyocyte models, will help to verify the BAG3 interactions and enable investigations of the more focused phenotypic effects. Additionally, validation through co-immunoprecipitation (Co-IP) studies, although challenging to scale up and expensive for large-scale applications will be important for confirming selected candidate proteins of interest in human heart tissues and human-induced pluripotent stem cell-derived cardiomyocytes (hIPSC-CMs) to corroborate our findings and enhance their relevance to the human heart.

## Figures and Tables

**Table 1:** 50 hallmark gene sets in idiopathic and ischemic cardiomyopathies.

HALLMARK	SIZE	Idiopathic	NES	FDR q-val	Ischemic	NES	FDR q-val	Proteins
ADIPOGENESIS	176	case	1.11	0.420844	controls	−1.20	0.169207	DBT,TST,MDH2
ALLOGRAFT_REJECTION	197	controls	−1.05	0.394748	case	1.07	0.487176	RPS9,MAP3K7
ANDROGEN_RESPONSE	98	controls	−1.83	0.001006	controls	−1.90	5.31 × 10^−4^	SRP19,SGK1
ANGIOGENESIS	35	case	1.21	0.27238	case	1.24	0.252174	
APICAL_JUNCTION	178	controls	−1.07	0.375625	controls	−1.02	0.461531	LIMA1,GTF2F1,EPB41L2,TSC1
APOPTOSIS	157	controls	−1.51	0.018923	controls	−1.40	0.044813	LMNA,ANXA1,CASP1,CASP4,CASP3,PPT1,BID,BNIP3L,SPTAN1
BILE_ACID_METABOLISM	99	case	1.74	0.009496	case	1.62	0.01886	PRDX5,ATXN1
CHOLESTEROL_HOMEOSTASIS	61	controls	−1.32	0.074947	controls	−1.28	0.104645	ANXA5
COAGULATION	134	case	1.42	0.075674	case	1.59	0.018179	ANXA1,CSRP1
COMPLEMENT	186	controls	−1.44	0.028868	controls	−1.33	0.0762	CASP1,CASP4,CASP3,ANXA5,PRSS3,CSRP1
DNA_REPAIR	135	controls	−0.93	0.655048	controls	−1.01	0.458158	REV3L,EDF1,CETN2,NME3,GTF2F1,RFC2,RFC3,HPRT1,TP53
E2F_TARGETS	187	controls	−1.57	0.012377	controls	−1.50	0.026689	RFC2,RFC3,PSIP1,CBX5,NUP153,PNN,TMPO,STMN1,NASP,RACGAP1,TP53
EPITHELIAL_MESENCHYMAL_TRANSITION	192	case	1.53	0.031229	case	1.82	0.005753	TAGLN
ESTROGEN_RESPONSE_EARLY	198	controls	−1.58	0.012051	controls	−1.64	0.006863	DHRS2,SVIL
ESTROGEN_RESPONSE_LATE	199	controls	−1.49	0.019649	controls	−1.49	0.025326	TST,HPRT1,DHRS2,SGK1
FATTY_ACID_METABOLISM	145	case	1.09	0.438963	controls	−1.15	0.224652	MDH2,ACADVL,UROD,MIF
G2M_CHECKPOINT	195	controls	−1.52	0.017902	controls	−1.48	0.027784	NCL,SFPQ,TMPO,STMN1,NASP,TOP1,KIF23,TPX2,RACGAP1
GLYCOLYSIS	186	case	1.02	0.49266	case	1.16	0.35265	MDH2,STMN1,NASP,MIF,PGK1,PC
HEME_METABOLISM	194	case	1.02	0.524825	case	0.98	0.637137	UROD,TOP1,CAST,YPEL5,HEBP1,BNIP3L,HDGF,PC,ASNS
HYPOXIA	192	controls	−1.77	0.002004	controls	−1.56	0.013079	NAGK,BNIP3L,PRDX5,MIF,PGK1,FOSL2,KLF6
IL2_STAT5_SIGNALING	177	controls	−1.50	0.019949	controls	−1.45	0.030848	CASP3,KLF6,PHLDA1
IL6_JAK_STAT3_SIGNALING	86	controls	−1.84	8.15 × 10^−4^	controls	−1.62	0.00786	IRF9
INFLAMMATORY_RESPONSE	194	controls	−1.83	0.001168	controls	−1.69	0.004573	KLF6
INTERFERON_ALPHA_RESPONSE	80	case	1.73	0.007606	case	1.78	0.004291	CASP1,IRF9,TRIM21,IFI44,SP110,IFIH1
INTERFERON_GAMMA_RESPONSE	177	case	1.27	0.201025	case	1.41	0.070512	CASP1,CASP4,CASP3,IRF9,TRIM21,IFI44,SP110,IFIH1
KRAS_SIGNALING_DN	195	case	0.86	0.829231	case	0.87	0.871536	KMT2D,SGK1
KRAS_SIGNALING_UP	196	controls	−1.06	0.38636	case	1.01	0.630874	WDR33
MITOTIC_SPINDLE	181	controls	−1.21	0.160671	controls	−1.25	0.127236	PCNT,CAPZB,PCM1,RANBP9,CD2AP,EPB41L2,TSC1,SPTAN1,KIF23,TPX2,RACGAP1,APC
MTORC1_SIGNALING	193	controls	−2.35	0	controls	−2.31	0	HPRT1,PGK1,PSMC2,ATP6V1D,ASNS
MYC_TARGETS_V1	189	controls	−2.18	0	controls	−2.27	0	HPRT1,PGK1,HNRNPC,EEF1B2,GLO1,C1QBP,PWP1,SF3A1,SERBP1,MRPL9,HDGF,CBX3,PHB2
MYC_TARGETS_V2	55	controls	−2.16	0	controls	−2.13	0	CBX3,TCOF1
MYOGENESIS	197	case	1.39	0.080993	case	1.21	0.276254	SVIL,SPTAN1,FHL1,TAGLN,PC
NOTCH_SIGNALING	28	case	0.91	0.762965	controls	−0.77	0.923318	PPARD
OXIDATIVE_PHOSPHORYLATION	194	case	1.82	0.004724	case	1.45	0.059126	MDH2,ACADVL,ATP6V1D,PHB2,NDUFB3,OPA1,NDUFB6,NQO2,NDUFV1
P53_PATHWAY	195	controls	−1.61	0.01035	controls	−1.47	0.028355	CASP1,DNTTIP2,TP53
PEROXISOME	96	case	1.18	0.309822	case	0.99	0.658104	PRDX5,VPS4B,ATXN1
PI3K_AKT_MTOR_SIGNALING	100	controls	−1.79	0.001342	controls	−1.68	0.004535	CFL1,ECSIT,PIKFYVE,MAP3K7
PROTEIN_SECRETION	95	controls	−1.28	0.096338	controls	−1.39	0.04544	PPT1,SNX2,ARFGAP3,VPS4B
REACTIVE_OXYGEN_SPECIES_PATHWAY	44	controls	−1.63	0.008859	controls	−1.63	0.007414	PDLIM1
SPERMATOGENESIS	124	case	0.84	0.81949	case	0.72	1	PEBP1,ZC3H14
TGF_BETA_SIGNALING	51	controls	−1.59	0.011077	controls	−1.68	0.005039	APC,MAP3K7
TNFA_SIGNALING_VIA_NFKB	195	controls	−2.50	0	controls	−2.26	0	NFKB2,TRIP10,SGK1,FOSL2,KLF6,PHLDA1,IFIH1
UNFOLDED_PROTEIN_RESPONSE	108	controls	−2.20	0	controls	−2.28	0	PARN,BAG3,FUS,EIF4A3,DCP2,KHSRP,ASNS
UV_RESPONSE_DN	140	case	1.09	0.409433	case	1.11	0.41775	SIPA1L1,RND3,PHF3,ATXN1
UV_RESPONSE_UP	155	controls	−1.98	0	controls	−1.96	0	TST,CASP3,PPT1,BID,UROD,ASNS,EIF5,CDC5L
WNT_BETA_CATENIN_SIGNALING	37	controls	−0.87	0.783655	controls	−0.88	0.759333	TP53,PPARD
XENOBIOTIC_METABOLISM	187	controls	−1.41	0.035226	controls	−1.47	0.028478	HPRT1,PC,PPARD
APICAL_SURFACE	34	controls	−0.94	0.655474	controls	−1.00	0.471533	
HEDGEHOG_SIGNALING	36	case	1.03	0.541695	case	0.96	0.646984	
PANCREAS_BETA_CELLS	40	controls	−0.53	0.998829	case	0.57	0.998143	

**Table 2:** Fifteen hallmark gene sets with at least thee BAG3-interacted proteins.

Gene Set	Note	BAG3-Interacted Proteins
Down-regulated in cardiomyopathies		
MYC_TARGETS_V1	Signal DNA damage-induced apoptosis [20]	HPRT1,PGK1,HNRNPC,EEF1B2,GLO1,C1QBP,PWP1,SF3A1,SERBP1,MRPL9,HDGF,CBX3,PHB2
E2F_TARGETS	Integrate cell cycle progression with DNA repair, replication, and G2/M checkpoints [21]	RFC2,RFC3,PSIP1,CBX5,NUP153,PNN,TMPO,STMN1,NASP,RACGAP1,TP53
G2M_CHECKPOINT	Prevent DNA damaged cells from entering mitosis [22]	NCL,SFPQ,TMPO,STMN1,NASP,TOP1,KIF23,TPX2,RACGAP1
P53_PATHWAY	Promote cell cycle arrest to allow DNA repair or apoptosis [23]	CASP1,DNTTIP2,TP53
APOPTOSIS	Cardiomyocyte apoptosis-interruptus [24]	LMNA,ANXA1,CASP1,CASP4,CASP3,PPT1,BID,BNIP3L,SPTAN1
HYPOXIA	Alter myocardial gene expression and induce cardiomyocyte apoptosis [25]	NAGK,BNIP3L,PRDX5,MIF,PGK1,FOSL2,KLF6
XENOBIOTIC_METABOLISM	Oxidative stress with excessive production of reactive oxygen species (ROS) [26]	HPRT1,PC,PPARD
UV_RESPONSE_UP	DNA damage repair [27]	TST,CASP3,PPT1,BID,UROD,ASNS,EIF5,CDC5L
UNFOLDED_PROTEIN_RESPONSE	Decrease global protein synthesis, and increase refolding or degradation of misfolded proteins [28]	PARN,BAG3,FUS,EIF4A3,DCP2,KHSRP,ASNS
TNFA_SIGNALING_VIA_NFKB	Cardioprotective in hypoxic or ischemic myocardial injury [29]	NFKB2,TRIP10,SGK1,FOSL2,KLF6,PHLDA1,IFIH1
MTORC1_SIGNALING	Regulate cell growth and metabolism [30] and mediate adaptive cardiac hypertrophy [31]	HPRT1,PGK1,PSMC2,ATP6V1D,ASNS
ESTROGEN_RESPONSE_LATE	Prevent apoptosis and necrosis of cardiac and endothelial cells [32]	TST,HPRT1,DHRS2,SGK1
PI3K_AKT_MTOR_SIGNALING	Lead to cell proliferation [33] and inhibit cardiomyocyte apoptosis [34]	CFL1,ECSIT,PIKFYVE,MAP3K7
IL2_STAT5_SIGNALING	Modulate CD4+ Th cell differentiation [35] and upregulate the expression of c-Myc, BCL-2, and BCL-x [36]	CASP3,KLF6,PHLDA1
Up-regulated in cardiomyopathies		
INTERFERON_ALPHA_RESPONSE	Interferon inhibits cardiac cell function in vitro [37] and interferon treatment has been shown of cardiotoxicity [38].	CASP1,IRF9,TRIM21,IFI44,SP110,IFIH1

## Data Availability

Supporting data from this study can be obtained by emailing the corresponding author Hakon Hakonarson.

## Supplementary Materials

The following supporting information can be downloaded at: https://www.mdpi.com/1422-0067/25/20/11308/s1.
