# Supplementary material for: The Role of BAG3 Protein Interactions in Cardiomyopathies
Source: Int J Mol Sci. 2024 Oct 21;25(20):11308. doi: 10.3390/ijms252011308 (PMC11605229; doi:10.3390/ijms252011308)
Supplement: Supplementary file 1 [file ijms-25-11308-s001.zip › supplementaryFigs.pdf]

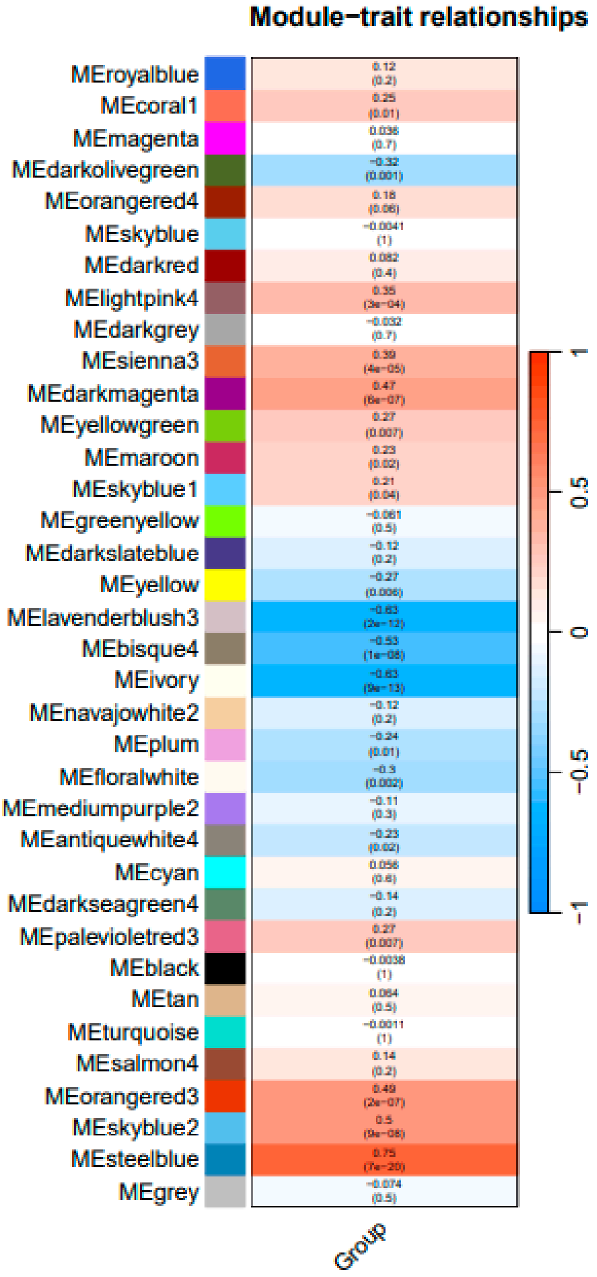

Figure S1 WGCNA module relationships in idiopathic cardiomyopathies. In 86 idiopathic cardiomyopathies, the gene module Module_ivory containing BAG3 is significantly downregulated (P value=9E-13). The genes in Module_ivory include: A2M; AHCY; AHSA1; ARL6IP5.1; ATP1A1; ATP2A2; BAG3; BBS1; BCL2L1; BCL2L1.2; BOP1 /// MIR7112;

BRD4.1; BTG2; CALU.2; CAST.1; CDK2AP2; CRYAB; CSNK1D; DAPK3 /// MIR637; DDX17.1; DNAJB5; EHD1.1; EIF3B.2; EIF3I; EPB41L1.1; ETS2.1; FADS3; FADS3.1; FGF22; FKBP4; FKBP9; FNDC3A.1; FOXK2; FUCA1; GADD45A; GGT5; GLT8D1; GM2A.1; GM2A.5; GOSR2; HIST2H2AA3 /// HIST2H2AA4; HIST2H2AA3 /// HIST2H2AA4.1; HSP90AB1; HSPA5; HSPA8 /// SNORD14C /// SNORD14D; HSPB1; HSPB8; HSPH1.1; HYAL2; HYOU1; IFI16.1; IMP4; ITGB5; ITM2B; KLF6; KRT8; MAP3K6; MCM2; MGAT2.2; MIR4745 /// PTBP1.3; MIR5193 /// UBA7.1; MLLT3; MPDU1; MYO9B.2; NDRG3; NFKBIB; NFKBIB.1; NOLC1; NOS3; NSDHL; OSMR; PAMR1; PDCD11; PDIA3; PDLIM5.3; PI4K2A; PLXNA2.1; POGZ; POR; PSMC4; PSMD11.1; PSMD8; PVR.1; PVR.3; R3HDM2; R3HDM4; RAB5C.1; RANGAP1; RBFOX2.3; RBM8A.3; RNF126; RRS1; SDF2L1; SEC13; SEPP1; SH3BGR; SHMT2.2; SLC52A2.1; SLC7A6; SRM; SRPK2.2; ST5; STIP1.1; TES; TM9SF1; TMEM208; USP32 /// USP6; VARS.1; WDR1.1; WDR18; WDR46; WDR74.1; YKT6.1.

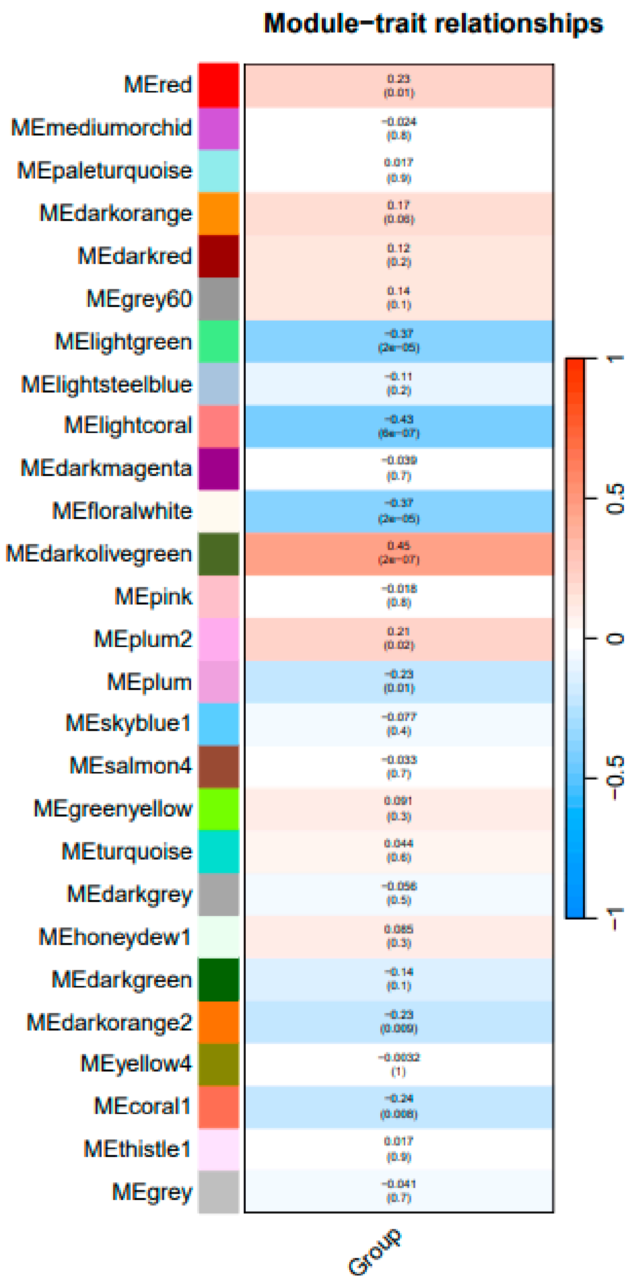

Figure S2 WGCNA module relationships in ischemic cardiomyopathies. In 108 ischemic cardiomyopathies, the gene module Module_ floralwhite containing BAG3 is significantly downregulated (P=2E-05). The genes in Module_ floralwhite include: MARCH3; MARCH5; SEP15; ABCE1; ABCE1.1; ABHD14A /// ACY1; ABHD5; ABHD5.1; ABHD5.2; ABR; ACKR4; ACLY.1; ACSL5; ACTR3.1; ACTR3.2; ADAM17.2; ADAM19; ADSL.1; AGBL5; AGFG1.2; AGFG1.3; AGO2 /// CASC7 /// CASC7 /// DQ574852; AGPAT5; AIMP1.1; AK6; AKAP12; ALDOC; ANGPT2; ANGPT2.1; ANKLE2; APEX1; APOLD1 /// DDX47; AQP1.1; ARC; AREG; ARF4.1; ARF6.1; ARFGAP3; ARG2.1; ARID4B /// RBM34; ARL2; ARL4A; ARPC3; ATF3; ATG12; ATG5; ATP6V0E2; AVL9.1; B4GALT5.1; BACH1; BAG3; BCAS3;

BCCIP; BCL3 /// MIR8085.1; BCL6; BDNF; BID.1; BIRC3; BRIX1; BTG3; BTG3.1; BUB3.3; BZW1.1; C12orf5; C1orf123; C20orf24 /// TGIF2-C20orf24; C2CD2; C3orf52; CA11; CACYBP; CACYBP.2; CBFB; CCDC47; CCDC86; CCNC; CCNL1; CCS; CCT2.1; CCT4; CCT5; CCT6A; CCT6A.1; CCT8; CD164; CDC37L1; CDK17.1; CDKN1A; CEBPD.1; CEBPG; CEBPZ; CFDP1; CHIC2; CHORDC1; CHST2; CLIC2; CLMN.1; CNIH4; CNOT4.1; CREM.3; CRYL1; CSGALNACT2; CTSO; CUX1; CXCL1; CXCL8; CXCL8.1; CYLD.2; CYR61; CYR61.1; DAAM2; DAZAP1; DDAH1; DDR1 /// MIR4640.1; DDR1 /// MIR4640.2; DDR1 /// MIR4640.3; DDX18.3; DDX39A; DERL2; DGKD; DGUOK; DGUOK.1; DHCR24; DHRS1; DHRS3; DHX15; DIEXF.1; DIEXF.2; DIMT1; DIMT1.3; DKC1 /// MIR664B /// SNORA56.1; DKK1; DLG5; DNAJA1; DNAJA1.1; DNAJB1; DNAJB1.1; DNAJC2; DNALI1; DNTTIP2; DUSP5; DUSP6; EBNA1BP2 /// MIR6733; ECT2; EDNRB; EDNRB.1; EDNRB.2; EEF1E1; EIF1AX; EIF2AK3; EIF2S1.2; EIF2S2.1; EIF3J.1; EIF4A3; EIF5.4; ELL2.1; ELOVL5; ELOVL6; ELOVL6.1; ELTD1; EMP1; EMP1.2; ENAH; EPHA2; ETF1; ETF1.1; EZH1; FAM127A; FAM216A; FAM46C; FBXL14; FEM1B; FERMT2.1; FGF2; FLAD1.1; FLI1; FLNC; FOSL1; FOSL2.1; FOXK2; FTSJ1; FXYD1; GABPB1; GABPB1.1; GADD45B; GADD45B.1; GADD45B.2; GAR1; GART.1; GAS6; GCH1; GCLM; GFPT2; GLRX2; GMPS; GNB5.2; GNG12; GNL2; GNL3 /// SNORD19B; GOLT1B; GPR56.1; GTF2B; GTPBP4.1; GTPBP4.2; H2AFY; H2AFZ; H2AFZ.1; HAMP; HBEGF; HBEGF.2; HCFC1R1; HEATR1; HEMK1; HERPUD1; HIF1A; HK2; HMGN2; HN1L.1; HNRNPD.3; HNRNPR.1; HOPX; HSP90AA1; HSP90B1 /// MIR3652.1; HSPA13; HSPA14; HSPA1A /// HSPA1B.1; HSPA1A /// HSPA1B /// HSPA1L; HSPA4.1; HSPA4.2; HSPA6; HSPA6.1; HSPD1.1; HSPE1; HSPH1; IARS; IFRD1; IFRD1.1; IL1RAP; IL1RAP.1; IL1RL1; IL24; IL6; IMP4; INSIG1.1; IPO4; IPO7.3; IQCG; IRAK3; IRS2; IRS2.1; ISG20L2.1; ITGB3.2; ITSN1.1; JMJD6; JMJD6.1; JTB; JUN.2; KIAA0020; KIAA0226; KIAA0247; KIF1B; KLF10; KLF5; KLHL2; KPNA2; KPNA2.1; LDB3; LDLR.1; LINC00312; LIPG; LMCD1; LOC100129518 /// SOD2.1; LOC100996792 /// MAP2K3.1; LRRC16A; LRRC23; LRRC59; LRRC8B.1; LSG1; LSG1.1; MAFF; MAFF.1; MAK16; MALT1; MALT1.2; MAP2K1; MAP3K7CL; MAPK6; MAPKAPK5; MAPRE1.1; MARS /// MIR6758.1; MBD2; MCM4.3; MED28; MET; METTL22; MFAP3; MGAT2.1; MID1IP1; MINA.1; MIR1292 /// NOP56 /// SNORD110 /// SNORD57 /// SNORD86.1; MIR1304 /// SNORA1 /// SNORA18 /// SNORA32 /// SNORA40 /// SNORA8 /// SNORD5 /// TAF1D; MIR21 /// VMP1; MIR22 /// MIR22HG; MIR3658 /// UCK2; MIR636 /// SRSF2.1; MIR636 /// SRSF2.2; MIS12; MORF4L2; MPHOSPH10; MSANTD3-TMEFF1 /// TMEFF1; MSX1; MTHFD2; MYC; MYH14.2; MYOT; MZF1.1; NAA50; NAMPT; NAMPT.1; NAV3; NDEL1; NECAB3; NETO2; NFE2L1.1; NFE2L1.2; NGDN; NIP7; NIPBL.3; NME1; NME4; NMT1; NOC3L; NOL11; NOLC1.2; NOP16; NOP16.3; NRBF2; NRIP1; NSMAF; NUP205; NUPL1; NXT1; OAZ2.1; OLFML2A; ORMDL2; P2RX5; PAICS; PAICS.1; PAK1IP1; PAMR1; PANX1; PAPOLA.1; PDCD2.1; PDIA6.1; PDPN; PDPN.2; PELI1; PFAS; PGM3; PGM3.1; PHLDA2.1; PICALM.1; PICALM.2; PIGA; PIGT; PIK3IP1.1; PITPNC1; PLCXD1; PLEKHA6; PLIN2; PLSCR1.1; PNO1; PNP; POLR2D; POLR3C; PPA1; PPP3CC; PPRC1; PRKCH.1; PRKCI.2; PRPF4; PRRC2B; PSMA5; PSMD12; PSMD14; PSMG1; PTEN /// PTENP1; PTGER3.9; PTGER4.1; PTGR2 /// ZNF410; PTP4A1.2; PTPRE; PTRH2; PTX3; PUS7; PVR; PXDC1; PYGB; RAB15.1; RAB22A.1; RAN; RAN.1; RASAL2.1; RBM3; RBM8A.1; RCAN1; RIPK2.1; RND3; RNF138; RNF19B; RNF4; RNMT.1; RPGR; RPL37A.1;

RRAS2.1; RRAS2.2; RRP1B.1; RRS1; RTN4.1; RUNX1T1.1; RYBP.1; SACS; SAR1A.1; SAT1; SEC24A; SEC24A.1; SEH1L; SEL1L; SELE; SERP1; SERPINB1; SERPINB1.1; SERPINE1; SERPINE1.1; SFPQ.1; SGK1; SLBP; SLC19A2; SLC25A11.1; SLC25A32; SLC29A1.1; SLC48A1.1; SLC7A1.3; SLIT3; SMARCA4.1; SMARCA4.4; SMEK2; SMURF2; SNHG17; SNORA29 /// TCP1; SNRPA1; SNRPA1.2; SNRPB; SNRPD1; SNRPD1.1; SNRPE; SNRPG; SOCS3; SORT1.1; SPCS3; SPCS3.1; SPINK1; SPTSSA; SQLE; SRP72.3; SRPK2.1; SRPK3; SRPRB; SRSF1.3; SRSF3.1; SRSF7.3; SS18L2; SSH1; SSR3; STK17A; STK17A.1; STK17A.2; STK3; STK38; STX12; STX12.1; TAF1B; TARDBP; TBCCD1; TBL2.1; TCEA2; TCEB1; TCEB1.1; TDG; TEAD4.1; TES.1; TFPI2.1; TGFB2.1; TGFB2.3; TGIF1; THBD; THBS1.1; THBS1.2; THBS1.3; TIAL1.1; TMA16; TMED5; TMEM165; TMEM5.1; TMEM70; TMEM70.1; TMX1.1; TNFAIP1; TNFAIP3.1; TNFAIP6; TNFAIP6.1; TNFRSF10B.1; TNFRSF11B; TNFRSF11B.1; TP53BP2; TPCN1; TRA2B.1; TRAFD1; TRAFD1.1; TRIB1; TRIO.3; TRMT5; TSPAN13; TUBB2A; TUBB6; TVP23B; TXN; TXN.1; TXNRD1; U2AF1; U2AF2.1; UAP1; UBA2; UBIAD1; UCHL3; UFD1L; UFM1; UGCG.1; UGDH; URB2; USP12; UTP18; UTP3; UTP6; VWF; WDR3; WDR43; WEE1; WFS1; WTAP; XBP1; XPOT; XRCC4; XRCC4.1; XRCC4.3; YES1.1; YRDC; ZMYND8.1; ZNF146; ZNF259P1 /// ZPR1; ZNF76; ZNF767P.
